# The Potential Use of Honey as a Neuroprotective Agent for the Management of Neurodegenerative Diseases

**DOI:** 10.3390/nu15071558

**Published:** 2023-03-23

**Authors:** Mohammad Adi Mohammad Fadzil, Suraiami Mustar, Aswir Abd Rashed

**Affiliations:** Nutrition Unit (NU), Nutrition, Metabolism and Cardiovascular Research Centre (NMCRC), Institute for Medical Research (IMR), National Institutes of Health (NIH), Ministry of Health Malaysia (MOH), No. 1, Jalan Setia Murni U13/52, Seksyen U13 Setia Alam, Shah Alam 40170, Malaysia

**Keywords:** honey, neurodegenerative, Alzheimer’s disease, Parkinson’s disease, polyphenols, antioxidant, anti-inflammation, anti-cholinesterase, in vitro, in vivo

## Abstract

As the global population ages, there is an increasing research on managing neurodegenerative diseases that mainly affect the elderly. Honey is one of the natural products and functional foods widely studied for its neuroprotective properties. This review investigates honey’s effectiveness as a neuroprotective agent through in vitro, in vivo, and clinical research. The articles were browsed from three databases (PubMed, ScienceDirect, and Scopus) between the years of 2012 and 2022 using the keywords “honey” crossed with “neurodegenerative”. Out of the 16 articles, six in vitro, eight in vivo, one combination study, and one clinical intervention were compiled. Among the various types of honey studied, the Tualang and Thyme honey exhibited the highest antioxidant, anti-inflammatory, and anticholinesterase activity, leading to the prevention and management of multiple neurodegenerative diseases such as Alzheimer’s disease. The neuroprotective properties of honey are primarily attributed to its high polyphenol content, with quercetin and gallic acid being the most prominent. This review compiled considerable evidence of the anti-neurodegenerative properties of honey presented by in vitro and in vivo studies. However, more clinical intervention studies are required to support these findings further.

## 1. Introduction

Currently, the only treatments for neurodegenerative diseases are those that either slow the progression of the disease or control its symptoms. Substantial numbers of studies have established the production of reactive oxygen species (ROS) as the cause of oxidative stress, leading to neurodegenerative diseases [1]. One of the contributing elements to the dysregulation of neurological activities is the difference between pro-oxidant and antioxidant cellular mechanisms that leads to mitochondrial damage, lipid peroxidation, neuroinflammatory processes, and endogenous dopamine metabolism [1,2]. Many scientists have been looking for compounds that activate ROS-blocking pathways or lessen their effects in recent years [3,4]. Significant research is being conducted to investigate and determine the availability of other prospective natural medications that are similarly effective and lack side effects to overcome the limits of current therapies available for neurodegenerative illnesses.

Alzheimer’s disease (AD) is the most common type of dementia. The pathophysiology of AD is mainly dominated by the amyloid cascade pathways [5]. Under normal healthy circumstances, amyloid precursor protein (APP) is cleaved by α-secretase and y-secretase. The by-products are then processed for clearance. However, in AD patients, APP is abnormally cleaved by β-secretase instead of α-secretase. The product of this abnormal cleavage is the insoluble Aβ (amyloid-beta) fragment. Aβ cannot be processed for clearance. Thus, it aggregates and accumulates outside of neurons, forming the pathological Aβ plaques. The tau protein maintains the structural integrity of microtubules in axons [5]. In AD patients, the tau protein is hyperphosphorylated, which causes microtubules to disintegrate. Then, the tau protein aggregates into neurofibrillary tangles, accumulating in the neurons. The iron hypothesis describes the abnormal influx of iron in the brain, which produces ROS and oxidative stress [5]. These proposed pathways mainly result in chronic inflammation, oxidative damage, and neuronal death [5]. The U.S. Food and Drug Administration (FDA) approved two main classes of drugs for AD, which are cholinesterase inhibitors (tacrine, donepezil, galantamine, rivastigmine) and N-methyl-D-aspartase (NMDA) receptor antagonists (memantine) [6]. In 2018, Dubey et al. reviewed the relationship between histone acetylation and its role in the expression of risk genes for AD. As people age, lower levels of histone acetylation were found due to the increased activity of histone deacetylases (HDACs). Studies using HDAC inhibitors showed increased histone acetylation, and the risk genes are better controlled, thus proposing HDAC to be a promising target for the epigenetic treatment of AD [6]. 

Parkinson’s disease (PD), which affects 2–3% of the old population, is the second most common neurological disease [7]. The hallmarks of the PD pathophysiology are the loss of dopaminergic neurons and the accumulation of intraneuronal misfolded alpha-synuclein proteins called Lewy bodies [8]. The accumulation of Lewy bodies and other dysfunctional proteins causes chronic inflammation in the brain and, ultimately, neuronal cell death. Gradual loss of the dopaminergic neurons in the substantia nigra results in dopamine deficiency, which is a neurotransmitter coordinating motor activity in the striatum. As a multifactorial disease, PD is affected by genetic and environmental risk factors. In a recent article using large-scale genome-wide association (GWASs), Nalls et al. identified 70 loci associated with PD. Many of these loci play a role in the pathways involving the clearance of dysfunctional and misfolded proteins [9]. Several risk genes with high penetrance have been associated with PD, as compiled by Simon et al., namely SNCA, PRKN, PINK1, and DJ-1 [8]. The conventional drugs for PD approved by the FDA are levodopa (L-DOPA), dopamine agonists, anticholinergics, catechol-O-methyltransferase (COMT) inhibitors, monoamine oxidase-B (MAO-B) inhibitors, and amantadine. These drugs only treat symptoms mainly by stimulating or inhibiting dopamine metabolism [10].

Codex Alimentarius (1981) defined honey as a “natural sweet substance produced by Apis mellifera L. bees from the nectar of plants, secretions of living parts of plants, or excretions of plant-sucking insects on the living parts of plants, which the bees collect, transform by combining with specific substances of their own, deposit, dehydrate, store and leave in the honeycomb to ripen and mature” [11]. In general, honey is also produced by other honeybee species (family Apidae, genus Apis), such as Apis dorsata and Apis cerana, which is stored in their honeycomb [12]. The stingless bees from the same family, Apidae, but genus Meliponini, also produce honey kept in a vessel in the beehives known as the honey pot [13].

Two honey varieties are available: blossom honey (nectar honey) and honeydew honey. Blossom honey is derived from the nectar of flowers, and honeydew honey is from the secretions of various parts of plants or plant-sucking insects (Hemiptera) on the living parts of plants [14]. Based on pollen content, blossom honey is categorized into unifloral (monofloral) and polyfloral multiflora). Unifloral honey has dominant pollen from one plant species, whereas polyfloral honey contains pollen from many different plant species. Unifloral honey has a unique taste and is priced higher than multifloral honey [15,16].

Honey is a highly concentrated sugar mixture primarily consisting of glucose (31%) and fructose (38%). Other disaccharides and trisaccharides include maltose, sucrose, isomaltose, gentiobiose, maltotriose, melezitose, isopanose, isomaltosylglucose, panose, and theanderose, which make up the remaining sugars [17]. In addition to carbohydrates, honey contains various other compounds that are beneficial to health and among them are polyphenols. Polyphenols, among others, are known for their antimicrobial, antiviral, antifungal, anticancer, antidiabetic, and protective effects on the digestive, respiratory, nervous, and cardiovascular systems [18,19]. 

Recent years have seen increased interest in functional foods due to consumers’ increased health concerns, which has sparked research into such foods [20]. Due to the excellent antioxidant potential of honey and other bee products such as royal jelly and propolis, they can be evaluated for their effects in disease prevention [21,22,23]. Functional food is often used in modern nutrition to define natural or processed foods that contain health-improving bioactive compounds. These bioactive compounds are believed to have the ability to boost the food’s usual known nutritional benefits [24]. A combination of different foods resulting in novel health benefits is also considered functional food [24]. In addition to fulfilling the body’s basic needs such as calorie consumption, consuming functional foods would provide the body with additional benefits such as cancer prevention, inflammation reduction, oxidative damage attenuation, and neurodegenerative protection [24]. 

Using honey to treat neurodegenerative diseases is becoming popular, since honey has long been consumed as a food supplement and shows no side effects if eaten in moderation. In comparison, using drugs to treat diseases often results in numerous complications due to side effects such as drowsiness, insomnia, confusion, and loss of concentration [25]. Investigations of honey’s influence on neurodegenerative disease prevention mainly focuses on the presence of polyphenol or phenolic compounds. Phenolic compounds have been thoroughly studied in the past. Thus, around 5000 phenolic compounds have been identified [26]. Phenolic acids and flavonoids are the most common phenolic compounds identified [26]. Various established cell models and polyphenols were used for neurodegenerative studies and enzyme analysis associated with the excretion of inflammatory cytokines and inhibiting enzymes that trigger cell inflammation responses. Polyphenols are also investigated for their potential to ameliorate the effects of dysfunctional proteins or pathological aggregates in the brain [27]. Recently, studies pivoted toward demonstrating polyphenols’ promising anti-neurodegenerative effects at the molecular level through epigenetic modifications, which may be the future of neuropathies treatment [28].

Several studies have looked into the perspective of using honey as preventive medicine for neurodegenerative disorders such as AD, amyotrophic lateral sclerosis (ALS), dementia, Huntington’s disease (HD), multiple sclerosis (MS), and PD.

## 2. Materials and Methods

**Figure 1:**
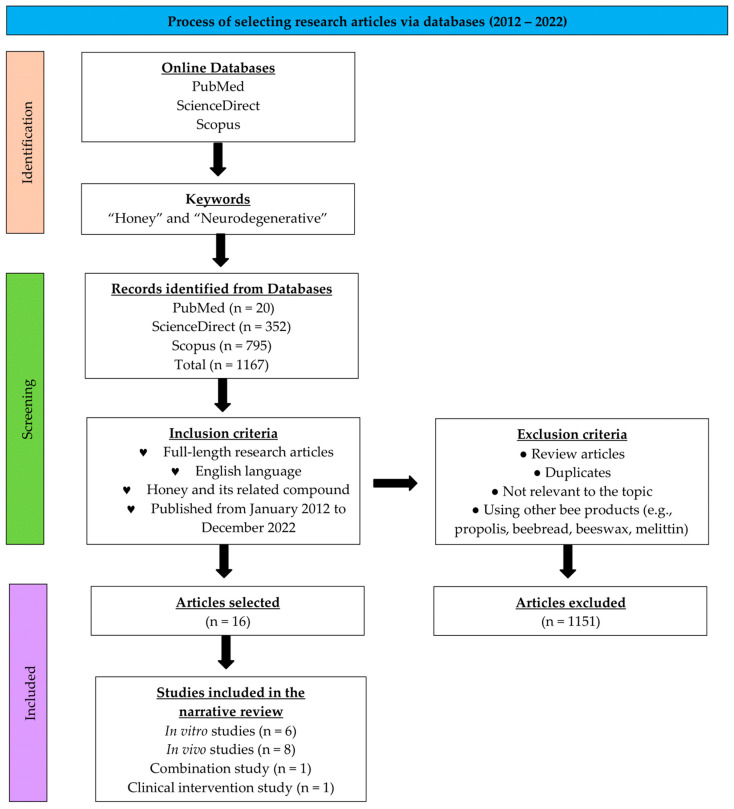
Flow diagram for the process of selecting research articles via online databases for the period of ten years (2012–2022).

Three databases—PubMed, ScienceDirect, and Scopus—were browsed for related articles. Keywords used for searching up articles were “honey” and “neurodegenerative”. Articles for this narrative review were only filtered for publication from 2012 to 2022 to preserve the recency factor of this review. Articles must be available in full English. Research articles written involving honey and its related compounds were included. Review articles and research articles using other products of bees, such as propolis, beebread, beeswax, and melittin, were excluded. Browsed articles were first filtered using title and abstract screening. Next, the filtered articles were finally subjected to full-text screening. Figure 1 shows the selection process using the inclusion and exclusion criteria.

## 3. Results

In the past ten years, only a few in vitro studies have been conducted on the beneficial use of honey for neurodegenerative diseases. Only six in vitro articles were found related to the search topic. Of the six in vitro honey studies, two in vitro articles [29,30] investigated AD and PD, whereas three in vitro articles [31,32,33] specifically targeted AD only. One in vitro study [34] investigated the relationship between honey and neurodegeneration. However, they did not specifically target any particular neurodegenerative disorder. Two in vitro studies investigated specific compounds present in honey, galangin [29] and apigenin [30]. A total of eight articles were found to use honey or its derivatives as preventive or therapeutic agents via in vivo investigations for various neurodegenerative disorders. Out of these, three in vivo studies [35,36,37] investigated the effect of honey on AD, whereas only one in vivo article [38] investigated the role of honey products on ALS. Mohd Sairazi and his team conducted two in vivo investigations [38,39] on the role of honey in curbing the effect of status epilepticus and other neurodegenerative disorders induced through excitotoxicity. The other two in vivo studies [40,41] investigated the relationship between honey and neurodegeneration. However, they did not specifically target a specific neurodegenerative disorder. It was interesting to note that four out of the eight in vivo studies evaluated the effects of Tualang honey (TUH) [35,39,40,42], while the other in vivo studies utilized Thyme honey (THH) [37], stingless bee honey (SBH) [41] and coffee honey [38]. One article by Campos et al. (2022) studied chrysin-a flavonoid found in honey, using both in vitro and in vivo techniques [43]. Lastly, only one intervention study [44] investigated the combination effects of honey and cinnamon on PD. The articles are compiled with their significant findings, as shown in Table 1, Table 2, Table 3 and Table 4. 

## 4. Discussion

In vitro investigations of honey’s influence on neurodegenerative disease prevention mainly focus on the presence of polyphenol or phenolic compounds. The earliest in vitro study within ten years until 2022 was in 2017 by Al-Abd and co-investigators. The study investigated the effect of galangin on neuroinflammation. Galangin is a polyphenol present in honey and propolis [29]. In the microglia BV2 cell line, galangin inhibited cytokines and NO. Microglia are brain immune cells rapidly initiated in traumatic brain injury and neurodegeneration by releasing various signaling pathways, ROS, inflammatory markers, and other substances [29]. Galangin inhibited NO, TNF-α, IL-6, and IL-10 creation in LPS-stimulated BV2 macroglia, which modulates neuroinflammation. Moreover, galangin showed no cytotoxicity effect on the cells at a concentration lower than 40 µg/mL. The results suggest that galangin may have potency as a prospective pharmaceutical medication or food supplement as a natural therapy for efficiently treating neuroinflammatory processes [29].

In 2018, a phenolic compound, apigenin, commonly found in honey and resveratrol in grapes and wine, was reported to induce neuron proliferation in murine N2a cells [30]. As known, neuronal deprivation is correlated with the onset of AD and PD. Therefore, further disease development can be controlled by enhancing neurons’ growth, survival, and differentiation. The results showed that polyphenols enhance neuronal differentiation and instigate neurite and axon growth, which imitates neurotrophic activity. In addition, apigenin showed no or minor cytotoxic effects compared to resveratrol and the control, retinoic acid. Thus, it may be further investigated as a potential neurodegenerative treatment [30]. 

The medications for AD are cholinesterase inhibitors, which inhibit AChE and BChE to increase the level of acetylcholine in the brain. AChE inhibitors are the preferred medications for treatment [45]. Zaidi and co-workers (2019) reported the suppression of AChE activity in honey with a percentage of inhibition ranging between 20.69 and 76.04%, with polyfloral honey showing the highest inhibition (76.04%) compared to monofloral honey (67.15%) [31]. The best IC50 value for AChE inhibition was 0.367 ± 0.025 mg/mL, which is not far from the control at 0.210 ± 0.020 mg/mL. Honey’s total polyphenols and flavonoid content correlate significantly with the AChE inhibitory efficacy. Although an AChE inhibitor is a leading choice to treat AD, it has various side effects that lead to new treatments targeting cholinesterase, AChE, and BChE. The inhibition of BChE has been reported to treat advanced AD [45]. A recent study assessed nineteen honey varieties’ inhibitory activity on AChE and BChE using Ellman’s colorimetric approach [33]. Thyme and Goldenrod honey significantly inhibit AChE and BChE with the highest degree of inhibition of 21.17% and 34%, respectively, raising the prospect that honey may be utilized as a future AD treatment. 

In the past ten years, few studies have used in vivo techniques to study the potential of honey against neurodegenerative disorders. The first in vivo study compiled from the past ten years was in 2014 by Al-Rahbi and the team. The study experimented on the roles of TUH on the cholinergic system and medial prefrontal cortex of ovariectomized rats subjected to stress routines [35]. Attenuated levels of estrogens in ovariectomized rats and chronic stress subsequently caused acetylcholine depletion in the cholinergic system [35]. Disruption of the cholinergic system in the medial prefrontal cortex is expressed in AD [35]. TUH managed to significantly increase the concentration of acetylcholine (*p* < 0.001) and decrease the concentration of AChE (*p* < 0.05) in the treated stressed ovariectomized rats. Other than that, histological comparisons showed significant improvement in the number and arrangement of Nissl-positive cells in the medial prefrontal cortex of the treated group compared to the untreated group. This study further proves the role of TUH as a phytoestrogen and a possible treatment for an impaired cholinergic system such as AD. 

The second and third in vivo studies were completed in 2017 and 2018 by Mohd Sairazi et al. to determine the effect of TUH against excitotoxicity induced by KA in rats’ brains [39,42]. In the 2017 study, Mohd Sairazi and their colleagues investigated the oxidative markers and histological assessment of KA-treated rats with TUH. KA is a neurotoxin often used in studies to induce excitotoxicity and neuroinflammation that models epileptic seizures [39]. KA treatment induces the accumulation of reactive oxygen species and increases oxidative stress, which is a significant source of neuronal destruction [39]. Thiobarbituric acid reactive substances (TBARS) assay measures the concentration of MDA, which is a biomarker of oxidative damage by lipid peroxidation [39]. Uric acid was measured to estimate total antioxidant status (TAS) [39]. The TBARS and TAS levels of the group pretreated with TUH showed significantly decreased (*p* < 0.05) lipid peroxidation activity induced by the administration of KA as well as significantly increased (*p* < 0.05) TAS compared to the untreated KA control. Cresyl staining was performed to examine healthy cells in the cortex, whereas Fluoro-Jade C (FJC) stain was used to mark degenerated neurons [39]. In comparison to the untreated KA control, there was a significant increase (*p* < 0.05) in the number of healthy cells and a decrease (*p* < 0.05) in the number of FJC-positive cells in the treatment group. This proves that TUH pre-treatment before the administration of KA caused attenuated neuron deaths and neurodegeneration in the cortex. 

The following year, in 2018, Mohd Sairazi et al. further investigated the role of TUH in attenuating neuroinflammation markers and caspase 3, an apoptotic marker in rat brain regions, after the administration of KA [42]. TUH treatment before the administration of KA attenuated the elevation of IL-1*β*, tumor necrosis factor-alpha (TNF-*α*), and COX-2 in the cerebral cortex, cerebellum, and brainstem (*p* < 0.05). The levels of AIF-1 protein and 5-lipoxygenase (5-LOX) were significantly attenuated in the cerebral cortex and cerebellum (*p* < 0.05). Caspases-3 were significantly attenuated in the cerebral cortex (*p* < 0.05), indicating that the pre-treatment of TUH successfully decreased cell death induced by KA [42]. One interesting finding was that the level of GFAP did not show any significant changes with the pre-treatment of topiramate, which is a GLuR5 kainate receptors inhibitor. However, the pre-treatment of TUH showed significant attenuation of GFAP in the cerebellum. These findings proved that TUH is a major candidate for adjunct AD treatment due to its neuroprotective ability.

Later in 2019, Asari et al. assessed the anti-neuroinflammatory characteristics of TUH and DHA-rich fish oil using chronic-stress-exposed rats. DHA is an omega-3 polyunsaturated fatty acid with anti-inflammatory properties [40]. The stress-exposed control groups showed increased levels of inflammatory markers such as TNF-*α*, IL-6, and IFN-g in the brain homogenates. These elevated levels of inflammatory markers were reduced in the groups treated with TUH and DHA-rich fish oil and those treated with the combination of TUH and DHA-rich fish oil. However, there were no significant differences between the three treatment groups. This indicates that TUH, DHA-rich fish oil, and their combination are equally potent in combating neuroinflammation.

The fifth in vivo study was conducted by Ranneh and colleagues in 2019. They investigated the neuroprotective effects of SBH on neuroinflammation and oxidative damage induced by LPS using rats [41]. Increased ROS and oxidative stress were indicated by the upregulation of the nuclear factor kappa-light-chain-enhancer of activated B cells (NF-κB) and p38 mitogen-activated protein kinase (MAPK) [41]. Nrf2 is a transcription factor that regulates the expression of antioxidants and several other defense pathways against oxidative stress [41]. SBH treatment decreased neuroinflammatory markers and downregulated the NF-κB and p38 MAPK pathways. In addition, SBH treatment upregulated antioxidant mechanisms by enhancing the Nrf2 pathways. In short, SBH can protect against neurodegeneration due to its anti-inflammatory and antioxidant properties. 

In 2020, Rosli et al. studied the role of MFF at 4 mL/kg against Aꞵ at 40 μg/200 μL concentration on rats using metabolomic 1H NMR spectroscopy analysis [36]. Key constituents of the commercial MFF utilized in this research were concoctions of pomegranate, date, and honey [36]. Twenty-nine metabolites were found in the tested groups. Due to the failure of principal component analysis (PCA) and partial least squares-discriminant analysis (PLS-DA) models to elucidate the significance of the metabolites, orthogonal projections to latent structures discriminant analysis (OPLS-DA) were performed. The OPLS-DA model significantly distinguished the metabolites among the studied groups; the model was validated using CV-ANOVA (*p* < 0.05). Twelve metabolites were identified with significant variable importance in projection scores, VIP > 0.7 and *p* < 0.05. The identified metabolites were then analyzed for their significance in metabolic pathways as possible biomarkers. Metabolic pathways analysis revealed that the identified metabolites were involved in amino acid and energy metabolisms [36]. One of the significant metabolites was leucine, which was elevated in the Aꞵ-42 group and decreased in the Aꞵ-42-MFF group. Leucine is a branched-chain amino acid (BCAA), in which its elevation is known to be related to neurotoxicity through microglia activation [36]. Other than that, the Aꞵ-42-MFF group had elevated levels of glutamine, which is known to have a neuroprotective effect due to its anti-inflammatory effects [36]. Pyruvate and lactate levels were reduced in the Aꞵ-42 group compared to the Aꞵ-42-MFF group. This would indicate that the MFF treatment improves neuronal energy production pathways, which combat the energy hypometabolism often prevalent in AD brains [36]. Other than that, the increased levels of 3-hydroxybutyrate in the Aꞵ-42-MFF group was another neuroprotection benefit of the MFF treatment because 3-hydroxybutyrate is known to increase neurons’ survivability against inflammatory and oxidative stresses [36]. The metabolomic assessment revealed that MFF-containing honey provided neuroprotection against Aꞵ-42 in rat brains by improving energy metabolism and amino acids. 

In 2020, Phokasem and the team conducted an in vivo study on a coffee honey product and its potential to reduce oxidative damage and improve learning and locomotive ability in dUbqn knockdown flies [38]. dUbqn mutations are associated with the early onset of ALS [38]. The accumulation of ROS has halved in coffee honey-treated dUbqn knockdown larvae compared to untreated dUbqn knockdown larvae. Corresponding to the ROS level, coffee honey-treated dUbqn flies showed reduced synapse structural defects, particularly in the number of boutons and size of terminal boutons compared to untreated dUbqn flies. The crawling distance of the coffee honey-treated dUbqn knockdown larvae was significantly longer than that of the untreated dUbqn knockdown larvae (*p* < 0.05, N = 20). The odor-taste assay was conducted to examine learning ability. The assay tested the ability of the larvae to choose the odor associated with rewards [38]. Coffee honey-treated dUbqn larvae made significantly more correct choices than untreated dUbqn larvae (*p* < 0.05). These findings conclude that coffee honey can reduce the damage inflicted by the depletion of dUbqn in the flies’ brains.

The most recent in vivo study found was completed in 2022 by Aameri et al. The study demonstrated the effectiveness of Iranian THH as a preventive and therapeutic agent in the AD rat model [37]. Preventive group rats received Iranian THH treatment and later were induced with AD using AlCl3 [37]. The therapeutic group was AD model rats subjected to Iranian THH treatment [37]. The Y-maze test, which is a trial to examine spatial memory loss [37], showed that both the preventive and the therapeutic groups scored high with no significant difference from the healthy control group. The total antioxidants for the preventive and therapeutic groups were significantly higher than the AD model group. One interesting finding was that the total antioxidant measured in the preventive and therapeutic groups was even higher than in the healthy control group. Excessive lipid peroxidation is detected in AD due to free radicals build-up and oxidative stress [37]. MDA concentrations in both the preventive and therapeutic groups were significantly lower than in the AD model group. Histological comparisons between the Iranian THH treatment group and the AD model group found significant improvement in the cell count and morphology of the cortex and different parts of the hippocampus. This study proved that Iranian THH possesses promising potential as a preventive measure and treatment of AD. 

Campos et al. (2022) tested chrysin, a honey flavonoid, against neurodegeneration using both in vitro and in vivo studies [43]. The antioxidant activity in vitro study used human neuronal SH-SY5Y cells induced with AlCl_3_, H_2_O_2_, or t-BuOOH to mimic neurotoxicity. Treatment with 4 μM of chrysin reached IC50 of ROS inhibition in SH-SY5Y cells induced with 100 μM of H_2_O_2_. Next, SH-SY5Y was treated with t-BuOOH (100 μM) to induce oxidative stress and later showed a reduction in oxidant activity with chrysin treatment (IC50 = 2μM of chrysin). However, only the concentration of 5 μM chrysin showed significant total antioxidant activity in the membrane-enriched fractions of neuronal SH-SY5Y cells. In addition, treatment with chrysin (5 μM) reduced oxidative stress (*p* < 0.05) and inflammatory cytokines, which include iNOS, IL-1B, and TNF-a (*p* < 0.05) in microglial THP-1 cells induced with LPS (1 μg/mL). In addition, chrysin (5 μM) attenuated the oxidative stress levels (*p* < 0.05) in neuronal SH-SY5Y cells induced with the Fenton reaction using FeSO_4_/H_2_O_2_ (50 μM/200 μM) and further catalyzed by AlC_l3_ (50 μM). Furthermore, chrysin (5 μM) treatment decreased the cytostatic effect (*p* < 0.05) and cell necrosis (*p* < 0.05) induced by AlCl_3_ in neuronal SH-SY5Y. In short, chrysin treatment reduced inflammatory and oxidative damage developed by AlCl_3_, H_2_O_2_, or t-BuOOH in neuronal SH-SY5Y and microglial THP-1 cells. For in vivo study, laboratory mice were treated with AlCl_3_-induced neurotoxicity to investigate the behavioral, biochemical, and histological changes implicated by chrysin treatment at 10, 30, and 100 mg/kg. An open-field test (OFT) and chimney test (CT) showed no improvement in the mice’s exploratory and locomotor activities despite chrysin treatment at various dosages. However, chrysin treatment in all dosage groups showed similar significant (*p* < 0.05) improvement in the nonspatial long-term memory evaluated using the step-down avoidance test (SDPAT). Both AChE and BChE levels were significantly normalized using chrysin in the hippocampus of AlCl3-treated mice. Significant differences were found between the tested groups in the hippocampal AChE (*p* < 0.05), BChE (*p* < 0.05), MDA (*p* < 0.05), carbonylated protein (CP) (*p* < 0.05), catalase (CAT) (*p* = 0.005), and superoxide dismutase (SOD) (*p* < 0.05) activities. On the other hand, significant differences were found between the tested groups in the brain cortex MDA (*p* < 0.05) and SOD (*p* < 0.05) activities. Chrysin treatment at all applied dosages showed similar significant ability in normalizing the hippocampal AChE, BChE, MDA, CP, CAT, and SOD activities. Similarly, chrysin treatment at all applied dosages showed equal significant ability in normalizing the brain cortex MDA and SOD activities. The histological assessment showed that chrysin treatment decreased neurodegeneration by AlCl3 in the hippocampus and cerebral cortex. Here, 100 mg/kg of chrysin proved to be the most effective in preventing the death of neurons. To sum up, Campos et al. provided substantial evidence of chrysin’s ability to prevent neurodegeneration and oxidative damage in AlCl3-induced mice. 

Even though several in vitro and in vivo studies have proven honey’s potential in preventing and treating neurodegenerative diseases, only one intervention study was found on this topic. In 2019, Kalia et al. wrote a case report on the implications of honey and cinnamon oral treatment on prolonging the on-time for a 71-year-old female clinically diagnosed with PD [44]. On-time was defined in the research as the duration of time in which the patient’s oral dopaminergic medications were able to prevent symptoms of PD such as stiffness, bradykinesia, and trembling [44]. Off-time was defined as when oral dopaminergic medications failed to prevent PD symptoms [44]. Due to the short on-time, the doctor had to increase the subject patient’s prescriptions dosage and frequency. However, despite the increase in drugs plus the aid of physiotherapy treatments, on-time failed to increase significantly. At the time of the intervention, the subject’s oral dopaminergic prescriptions were 200 mg of Entacapone BP, 100 mg of L-DOPA, and 35 mg of Carbidopa anhydrous, to be taken once every 3 h. Later, for eight weeks and two times a day, the individual was instructed to consume a mixture of 1/4 teaspoon crushed cinnamon and 1/2 teaspoon honey in addition to the drug prescriptions and standard physiotherapy treatment. At the end of the treatment, the subject’s on-time improved from 2 to 3.5 h. Although this was based only on an individual case report, it warrants larger-scale clinical trials to develop honey and cinnamon as complementary treatments for PD.

**Figure 2:**
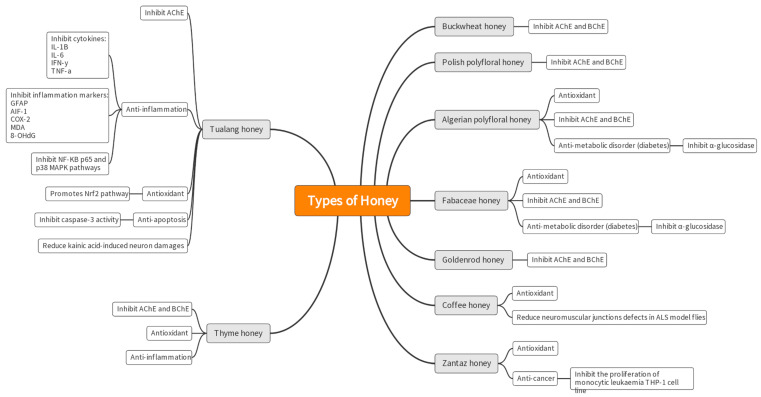
Summary of the types of honey and their potential for neuroprotection from the articles compiled.

Figure 2 shows a summary of the compiled articles on the types of honey and their potential as a neuroprotection agent. Some articles studied major compounds from various types of honey, whereas others utilized honey without specifying specific compounds in honey.

**Figure 3:**
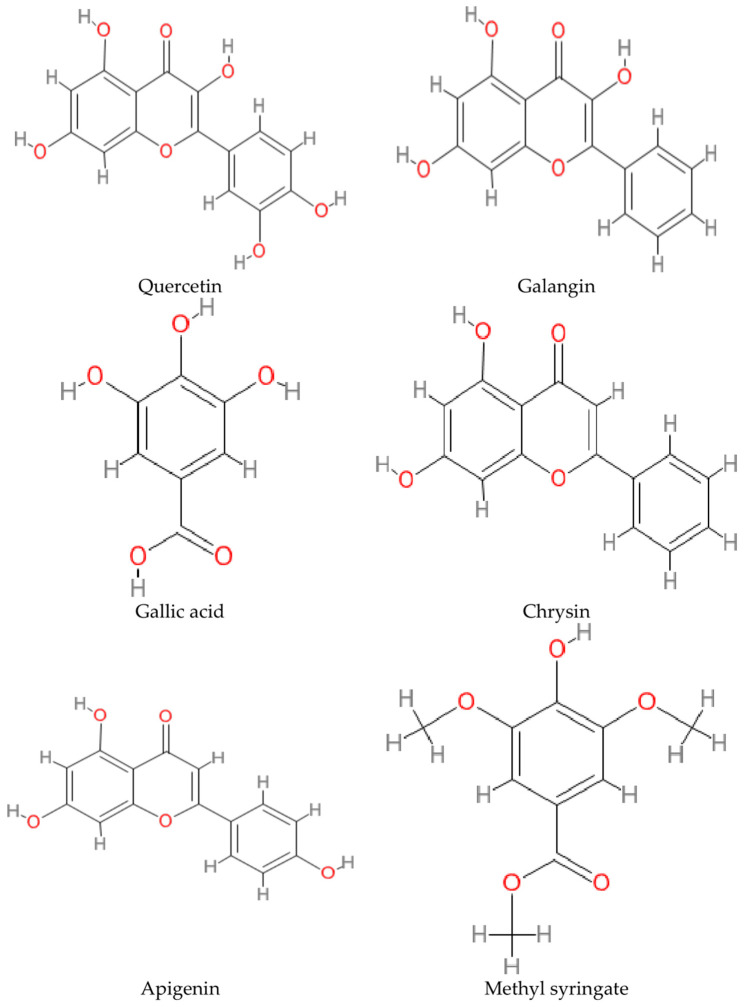
Chemical structures of the compounds from honey studied for anti-neurodegenerative properties.

The chemical structures of the bioactive phenolic compounds that play an essential role in influencing honey’s neuroprotective properties are depicted in Figure 3. In addition to the compounds investigated in the articles, other biologically active phenolic acids were also reported to be found in honey, such as cinnamic acid, coumaric acid, benzoic acid, chlorogenic acid, vanillic acid, and syringic acid. According to an HPLC study on Australian unifloral honey by Martos et al., five flavonoids are ubiquitous in all honey samples: myricetin, tricetin, quercetin, luteolin, and kaempferol [46]. Other flavonoids in honey include naringenin, hesperidin, fisetin, wogonin, genkwanin, acacetin, catechin, epicatechin, and pinobanksin-3-O-propionate [37,39].

**Figure 4:**
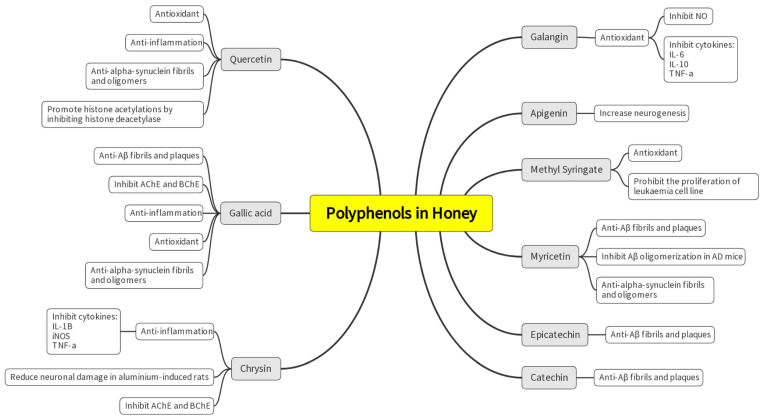
Highlighted polyphenols in honey and their potential for neuroprotection.

Other than their known antioxidant and anti-inflammation properties, polyphenols show great potential in dissolving pathological aggregates in the brain and inhibiting neurotoxicity from existing neuronal deposits. Freyssin et al. compiled in vitro studies elucidating the potential of polyphenols to attenuate the accumulation of many neurodegenerative disease-causing aggregates. The study highlighted the ability of catechin, curcumin, epicatechin, and myricetin to ameliorate the formation of amyloid fibrils and plaques. The highlighted polyphenols also showed the potential to reverse the formation of amyloid fibrils to their simpler oligomers [27]. This finding is supported by an in vivo study by Hamaguchi et al., which found that myricetin inhibited Aβ oligomerization in AD mice, thus preventing it from forming Aβ plaque. The authors concluded that myricetin is a promising agent for treatment targeting Aβ oligomerization [47]. In addition, Freyssin et al. compiled studies on multiple polyphenols that also showed potential in inhibiting the formation of alpha-synuclein fibrils and oligomers such as quercetin, myricetin, gallic acid, baicalein, and tannic acid. Although the exact mechanism is still being studied, the anti-aggregates properties of polyphenols may be attributed to their numerous derivatives configurations and moieties. Interaction between the polyphenol’s molecules with the protein’s β-sheets causes structural stability changes, which prevent the protein from hyperphosphorylation or spontaneous changes into its pathological forms [27]. Figure 4 shows the highlighted polyphenols and their potential as neuroprotection agents.

Based on the articles in this review, quercetin, and gallic acid were the most abundant flavonoid and phenolic acids found in honey. In support of that, upon assessing 40 commercial types of honey, Cheung et al. found that gallic acid was the most abundant phenolic acid. They also reported that the most abundant flavonoids were quercetin and luteolin [48]. In addition, quercetin and gallic acid found in TUH and THH were reported to contribute significantly to anti-neurodegenerative properties [37,39].

Quercetin is a natural pigment that belongs to the flavonoid group. Quercetin is ubiquitously found in natural products, including honey. Through extensive studies, quercetin has been proven numerous times in vitro and in vivo and clinical studies to show anti-neurodegeneration protection [49]. Although the precise mechanism of action contributing to its anti-neurodegenerative properties is still unclear, several promising theories exist. Quercetin is an antioxidant that can reduce oxidative species mainly due to two pharmacophores within the molecular structure that possess efficient arrangements for free radical scavenging [50]. The configuration involves a hydroxyl group at position 3 of the cyclic ring and a catechol group in the B ring [50]. Moreover, it also plays a role in enhancing the cells’ mechanisms of defense against oxidative damage. Quercetin can accomplish this by acting as a neuro-hormetic phytochemical. Quercetin, at a subtoxic level, can activate the Nrf2-antioxidant response elements (Nrf2-ARE) pathway. The Nrf2-ARE pathway is an adaptive response mechanism that controls various cellular antioxidant proteins [51]. Other than that, quercetin can stimulate the enzyme paraoxonase 2 (PON2) pathway, which contributes to neuroprotection against oxidative stress [49]. Furthermore, quercetin enhances the autophagy of misfolded proteins and malfunctioned organelles through the induction of sirtuins (SIRT1) [49]. A recent study elucidated the potential of quercetin dihydrate and 17 other bioactive compounds to modify histone acetylation in a heart model using bovine cardiac tissue [28]. Interestingly, quercetin proved to be the top two most potent inhibitors of HDAC. Due to its ability to inhibit most HDAC isoforms, quercetin is reported as a pan-HDAC inhibitor. This opens a new opportunity to utilize quercetin as an agent of epigenetic treatment by modifying histone acetylation and thus controlling the expression of neuropathies-related risk genes. These hypotheses support the neuroprotection capabilities of quercetin. 

Gallic acid is classified as phenolic acid, and it is commonly known for its bioactive and antioxidant properties. Gallic acid can be primarily found in plants and can also be found in honey [52]. Gallic acid possesses various derivatives with different pharmacological properties, such as hydrophobicity and molecular weight [52]. Gallic acid and its variation of derivatives allow it to target different pathways, resulting in various benefits [52]. Gallic acid’s anti-inflammatory property is mainly attributed to its potent ability to scavenge and neutralize ROS, making it a very reliable antioxidant [52]. Gallic acid can reduce neuronal damage caused by Aꞵ deposition by ameliorating the influx of Ca + 2 ions in the neurons, thus inhibiting glutamate release and the production of ROS [53]. In addition, gallic acid was proven to reverse the plaque-caused Aꞵ deposition, thus preventing synaptic and neuronal damage [54]. In a more recent study in 2014, Ardah et al. investigated the potential of gallic acid in inhibiting alpha-synuclein aggregation using an in vitro method. The study was conducted by using multiple physiochemical assays and cell viability assays. Gallic acid inhibited alpha-synuclein aggregation formation by dissolving premature alpha-synuclein amyloid fibrils, thus attenuating its neurotoxicity effects. Due to gallic acid’s multiple hydroxyl groups and its phenyl ring configuration, it could bind and stabilize soluble oligomers, preventing it from forming pathological insoluble aggregates [55]. Moreover, gallic acid is also a potent cholinesterase inhibitor, thus preventing neurotransmitter deficiency, which will lead to memory loss in neurodegenerative patients [56]. The following arguments support gallic acid’s neuroprotection properties.

## 5. Conclusions

Although we have found sixteen relevant studies around the world related to the health benefits of honey on neurodegenerative diseases, which comprise in vitro, in vivo, a combination of in vitro and in vivo, and intervention studies, they are still not sufficient to prove the effectiveness of honey in helping to control or reduce the progression of neurodegenerative diseases because only one intervention study has been conducted so far. However, we found that a positive role is played by the presence of active compounds in honey that help inhibit the secretion of certain enzymes and further help inhibit neurodegenerative progression. TUH and THH, as well as compounds such as quercetin and gallic acid, show the most promising potential as anti-neurodegenerative agents in terms of their potential negating neurodegenerative pathways. Thus, we can conclude that more research, with particular emphasis on intervention studies, is needed to increase knowledge and understanding, leading to findings that are anticipated to address these problems.

## Figures and Tables

**Table 1:** In vitro studies on using honey for the therapy of neurodegenerative diseases.

No.	Targeted Neurodegenerative Disease(s)	Key Findings	Reference
1.	AD, PD	-Galangin (flavonoid) inhibits nitric oxide (NO), interleukin 6 (IL-6), tumor necrosis factor-alpha (TNF-α), and interleukin 10 (IL-10) synthesis in lipopolysaccharide (LPS)-stimulated BV2 macroglia which modulates neuroinflammatory activity. -Galangin prevents neuroinflammation and could be a promising pharmacological agent or food supplement.	[29]
2.	AD, PD	-Apigenin is present in honey, parsley, rosemary, and olive oil, which are standard in the Mediterranean diet. -Resveratrol and apigenin were discovered to promote neuron development and imitate neurotrophic activity.	[30]
3.	AD	-Neurological problems in AD can be prohibited by inhibiting acetylcholinesterase (AChE). -Associations were found between antioxidants with total phenolic compounds and flavonoids content in selected Algerian honey samples. The samples also show anti-inflammatory, anti-AChE and anti-α-glucosidase activities. -The inhibition of AChE was reported to be in the range of 20.69 to 76.04% with IC50 values of 0.367 to 0.629 mg/mL. -Galantamine was used as a control for AChE inhibition and showed IC50 of 0.210 mg/mL, comparable to the honey test groups.	[31]
4.	AD	-This study used the colorimetric method to analyze 47 varieties of Polish honey as a source of AChE and butyrylcholinesterase (BChE) inhibitors. Buckwheat honey had the best potential to inhibit AChE (39.51% inhibition), while multifloral honey had the highest potential to inhibit BChE (39.76%). No IC50 values or drug control were mentioned in the article.	[32]
5.	Unspecified neurodegenerative disorder	-Zantaz honey contains methyl syringate (more than 50% of the total polyphenols), gallic acid, epicatechin, syringic acid, and catechin. -Using principal component analysis (PCA), methyl syringate and gallic acid showed the strongest positive correlation with antioxidant activities and cell proliferation inhibition in Caco-2 and THP-1 cells.	[34]
6.	AD	-Thyme, linden, bean, and heather honey showed high inhibition of AChE compared to the other 15 types of honey tested. Inhibition ranged from 18.31% to 21.17%. -Goldenrod, thyme, heather, buckwheat, and acacia honey inhibited BChE at a percentage ranging between 26% and 34%. There were no IC50 values, and no drug control was reported. -Positive correlations were shown between TPC and anti-AChE and anti-BChE activities.	[33]

**Table 2:** In vivo studies on using honey for the therapy of neurodegenerative diseases.

No.	Targeted Neurodegenerative Disease(s)	Methods/Key Findings	Reference
1.	AD	-Two months after ovariectomy, the rats were treated with 18 days of TUH or 17 β-estradiol as the control. During the last 15 days, the rats were subjected to stress routines and finally sacrificed. -The treatment of either TUH or 17 β-estradiol attenuated the reduction in acetylcholine and elevation of AChE in the brain homogenates of stressed ovariectomized rats. -The treatment of either TUH or 17 β-estradiol also showed a healthier order and number of Nissl-positive cells in medial prefrontal cortex (mPFC) neurons compared to untreated stressed ovariectomized rats.	[35]
2.	Unspecified neurodegenerative disorder	-Five groups of rats—normal saline, KA (kainic acid)-induced, TUH with KA-induced, anti-inflammation aspirin with KA-induced, and anti-epileptics topiramate with KA-induced. Five treatments were given for two and a half days. After the last treatment, KA was administered. -Pre-treatment of TUH reduced the locomotor hyperactivity, thiobarbituric acid, and neuronal degeneration induced by KA in the piriform cortex. -The TUH pre-treatment also reduced the weakening of the antioxidant status post-KA.	[39]
3.	Unspecified neurodegenerative disorder	-Rats were treated with TUH five times for the duration of two and half days. After the last treatment, the rats were induced with KA. Anti-epileptics topiramate was used as a control. -Pre-treatment with TUH for KA-induced status epilepticus rats significantly (N = 72, *p* < 0.05) attenuated the elevation of neuroinflammation markers such as TNF-*α*, interleukin-1 beta (IL-1*β*), glial fibrillary acidic protein (GFAP), allograft inflammatory factor 1 (AIF-1), and cyclooxygenase-2 (COX-2) level in the cerebral cortex, cerebellum, and brainstems. -The TUH pre-treatment also weakened the caspase-3 activity in the cerebral cortex.	[42]
4.	Unspecified neurodegenerative disorder	-Rats were subjected to stress routines and induced with TUH or docosahexaenoic acid (DHA)-rich oil or both. The duration was 28 days. -TNF-a, IL6, and interferon-gamma (IFN-y) concentrations in the brain homogenates of rats treated with DHA-rich fish oil, TUH, and TUH-DHA were lower than those in the control and stress-only-exposed groups (*p* < 0.05), but there was no difference across groups that received treatments.	[40]
5.	Unspecified neurodegenerative disorder	-LPS-induced-chronic subclinical systemic inflammation (CSSI) rats were treated with SBH. -Rats were administered LPS thrice every week for four weeks. Then, the rats were treated with SBH for one month. -The treatment significantly reduced inflammatory markers, MDA, 8-hydroxy-2′-deoxyguanosine (8-OHdG), NF-κB p65, p38 mitogen-activated protein kinase (p38 MAPK), and organ damage. -The treatment also enhanced antioxidants and nuclear factor erythroid 2–related factor 2 (Nrf2) expression.	[41]
6.	AD	--Rats were induced with Aβ-42 for 14 days to model AD. The rats were then given mixed functional food (MFF) treatment for 30 days. N-acetylcysteine was used as a control. -Aβ-induced rats were treated with MFF containing honey, dates, and pomegranate, which later improved the rats’ spatial memory and learning in the Y-maze test. -Metabolomic analysis using 1H NMR spectroscopy showed 12 metabolites that portrayed significant differences. Metabolic pathway analysis revealed that the MFF improved amino acid and energy metabolism, thus providing a neuroprotective effect.	[36]
7.	ALS	-ALS is associated with the human ubiquilin two genes. Ubiquilin (dUbqn) knockdown flies were treated with coffee honey. -Flies were cultured using standard fly-medium spiked with coffee honey (1% *v/v*). -Coffee honey (1% *v/v*) treatment proved the recovery of structural defects in neuromuscular junctions (NMJs), increased learning potential, and decreased the accumulation of ROS caused by dUbqn depletion in the brain.	[38]
8.	AD	-One group of rats received THH treatment for two weeks and then four weeks of THH accompanied by AlCl3 treatment to induce AD. Another group received six weeks of THH and AlCl3 treatment. Rivastigmine and tap water were used as control. -AD rats showed neurodegeneration, hippocampal damage, and increased malondialdehyde (MDA) and performed poorly in the Y-maze test. -Iranian THH increases the total oxidant, frequency of healthy cells, and normal neurons in all parts of the cortex and hippocampus. The behavior evaluation showed no significant difference between the effects of honey and the rivastigmine control group.	[37]

**Table 3:** A study using in vitro and in vivo on honey to treat neurodegenerative diseases.

No.	Targeted Neurodegenerative Disease(s)	Key Findings	Reference
1.	Unspecified neurodegenerative disorder	-In the in vitro study, neuronal SH-SY5Y cell lines treated with chrysin showed the capacity to mitigate the early oxidative stress induced by the oxidant tert-butyl hydroperoxide (t-BuOOH) which mimics lipid peroxidation. The IC50 for the ROS inhibition in neuronal SH-SY5Y cells were 4 μM chrysin/100 μM H_2_O_2_ and 4 μM chrysin/100 μM t-BuOOH. The treatment also counteracted the Fenton reaction in the presence of AlCl3 and the late necrotic death triggered by the reaction. -In the in vivo part, mouse models of neurotoxicity induced by chronic exposure to AlCl_3_ for 90 days were treated with chrysin. The mouse showed reduced cognitive impairment, and the hippocampus’s AChE and BChE activities were regulated. The treatment also reduced oxidative damage and necrotic cell frequency in the brain cortex and hippocampus.	[43]

**Table 4:** Intervention study on the use of honey for the therapy of neurodegenerative diseases.

No.	Targeted Neurodegenerative Disease(s)	Key Findings	Reference
1.	PD	-A 71-year-old female subject diagnosed with PD more than 15 years prior is the topic of this case report. Clinical improvement was seen with the cinnamon and honey therapy when it was shown to increase “on-time” with oral pharmaceutical medication. “On-time” was defined by the investigators as the duration when the PD drugs consumed by the patient could negate PD symptoms from re-emerging.	[44]

## Data Availability

Not applicable.

## Abbreviations

1H NMR	Proton Nuclear Magnetic Resonance
5-LOX	5-Lipoxygenase
8-OHDG	8-Hydroxy-2′-Deoxyguanosine
ACHE	Acetylcholinesterase
AD	Alzheimer’s Disease
AIF-1	Allograft Inflammatory Factor 1
ALS	Amyotrophic Lateral Sclerosis
APP	Amyloid Precursor Protein
BCHE	Butyrylcholinesterase
CAT	Catalase
COMT	Catechol-O-Methyltransferase
COX-2	Cyclooxygenase-2
CP	Carbonylated Protein
CSSI	Chronic Subclinical Systemic Inflammation
CT	Chimney Test
DHA	Docosahexaenoic Acid
dUBQN	Ubiquilin
FDA	U.S. Food and Drug Administration
FJC	Fluoro-Jade C
GFAP	Glial Fibrillary Acidic Protein
GWASs	Genome-Wide Associations
HD	Huntington’s Disease
HDACs	Histone Deacetylases
IFN-G	Interferon Gamma
IL-10	Interleukin 10
IL-1*β*	Interleukin-1 Beta
IL-6	Interleukin 6
KA	Kainic Acid
L-DOPA	Levodopa
LPS	Lipopolysaccharide
MAO-B	Monoamine Oxidase B
MDA	Malondialdehyde
MFF	Mixed Functional Food
mPFC	Medial Prefrontal Cortex
MS	Multiple Sclerosis
NF-ΚB P65	Nuclear Factor kappa B p6
NMDA	N-methyl-D-aspartase
NMJS	Neuromuscular Junctions
NO	Nitric Oxide
NRF2	Nuclear Factor Erythroid 2–Related Factor 2
OFT	Open-field Test
P38 MAPK	P38 Mitogen-Activated Protein Kinase
PCA	Principal Component Analysis
PD	Parkinson’s Disease
PON2	Paraoxonase 2
ROS	Reactive Oxygen Species
SBH	Stingless Bee Honey
SIRT1	Sirtuins
t-BuOOH	Tert-butyl Hydroperoxide
TAS	Total Antioxidant Status
TBARS	Thiobarbituric Acid Reactive Substances
TUH	Tualang Honey
THH	Thyme Honey
TNF-A	Tumor Necrosis Factor Alpha
